# Exploring Atypical Origins of Trismus: Surgical Solutions for Rare Pathologies—Insights from Rare Clinical Cases

**DOI:** 10.3390/diagnostics15111360

**Published:** 2025-05-28

**Authors:** Ioanna Kalaitsidou, Mathieu Gass, Dimitris Tatsis, Sherin Khalil, Christian Schedeit, Simon David Marjanowski, Sarah Wiegner, Benoît Schaller

**Affiliations:** 1 Department of Cranio-Maxillofacial Surgery, Inselspital, Bern University Hospital, University of Bern, CH-3010 Bern, Switzerland; mathieu.gass@insel.ch (M.G.);; 2 Department of Oral and Maxillofacial Surgery, Aristotle University of Thessaloniki, 54124 Thessaloniki, Greece; dtatsis@outlook.com

**Keywords:** trismus, osteoma, hyperplasia, myositis ossificans, osteomyelitis, abscess, ear canal

## Abstract

**Background**: Trismus, or restricted mouth opening, can present significant challenges in oral and maxillofacial surgery and trigger substantial functional and psychosocial disabilities. Intra-articular causes, such as temporomandibular joint ankylosis and arthritis, are thoroughly described; however, extra-articular pathologies like neoplastic, traumatic, infectious, and fibrotic conditions of adjacent soft and hard tissues are less frequently reported and present distinct diagnostic complexities and therapeutic hurdles. This retrospective study aims to investigate the difficulties encountered in diagnosis and surgical interventions associated with rare extra-articular causes of trismus. **Material and Methods**: This article describes five rare causes of extra-articular trismus. The cases range from benign pathologies like coronoid hyperplasia and osteomas to more complex diagnoses of myositis ossificans, external auditory canal abscess, and chronic osteomyelitis. A thorough diagnostic workup was performed for each patient, and specific surgical interventions were administered based on their pathology. **Results**: All five patients showed significant improvements in mouth opening after surgery. Diagnostic accuracy was ensured with advanced imaging modalities and innovative surgical techniques, and adequate postoperative care translated the favorable outcome. **Conclusions**: Although based on individual case descriptions, this study emphasizes the potential importance of early diagnosis, a multidisciplinary approach, and individualized treatment planning in managing rare extra-articular causes of trismus. These cases suggest a basis for a more organized system for the timely identification and treatment of such conditions. Additional research is needed to improve diagnostic accuracy, optimize surgical management, and develop evidence-based aftercare treatment to improve patient care and quality of life.

## 1. Introduction

Trismus, or restricted mouth opening, is a common problem encountered in oral and maxillofacial surgery [1]. The term originates from the Greek word “τρισμός” (trismos), meaning gnashing or grinding, and is described as a sustained, tetanic spasm of the mastication muscles that hinders normal jaw movement [2,3]. It is often considered both a symptom and a complication of temporomandibular disorders (TMDs), a collective term encompassing clinical conditions affecting the temporomandibular joint (TMJ), the masticatory muscles, and associated structures. TMDs are among the most prevalent musculoskeletal disorders in the craniofacial region, affecting approximately 31% to 34% of the population, with higher prevalence reported in women and individuals aged 20–40 years [4,5].

The normal range of mouth opening is variable, usually between 40 and 60 mm; an interincisal distance of less than 35 mm is often associated with severe functional disturbance [6]. Patients with trismus experience considerable difficulties in eating, speaking, and maintaining oral hygiene, which can severely impact their psychosocial well-being and quality of life [7].

The etiology of trismus has a wide range, including both intra-articular and extra-articular causes. Intra-articular pathologies, such as temporomandibular joint ankylosis or arthritis, are well-documented and often recognized early, whereas extra-articular causes are rare and often present a challenge for the clinician in the diagnosis process due to their low prevalence and heterogeneity. The uncommon causes involve neoplastic, traumatic, infectious, or fibrotic processes in the surrounding soft and hard tissues [2,8].

Knowledge of the underlying pathology, along with appropriate management and treatment, is essential, and prolonged treatable delay and inappropriate treatment lead to progressive functional decline, decreased quality of life, and worse prognosis [9,10]. The previously available literature on this topic is limited and primarily consists of isolated case reports, probably due to the uncommonness of extra-articular causes, despite the advancements in imaging and surgical techniques. This discrepancy highlights the importance of a holistic perspective on the diagnosis, categorization, and management of such infrequent diagnoses [10].

We describe several uncommon extra-articular causes of trismus and the challenges encountered while diagnosing and managing each entity. By presenting five rare clinical cases, we aim to contribute to understanding these unusual pathologies and enhance a systematic approach to their management. These conditions are individually infrequent, with the existing literature largely limited to isolated case reports or small case series. The scarcity of comprehensive epidemiological data reflects the inherent difficulty in studying such rare entities; nevertheless, their potential to cause significant functional impairment underscores the importance of early recognition and individualized treatment.

We hypothesize that such rare extra-articular causes of trismus are frequently underrecognized and that their structured presentation can contribute to earlier diagnosis and more effective treatment strategies.

## 2. Materials and Methods

A retrospective review was performed on patients diagnosed with rare extra-articular causes of trismus, treated at the Department of Oral and Maxillofacial Surgery from January 2018 to December 2024. The inclusion criteria discussed are those of cases with reduced mouth opening (defined as an interincisal distance of less than 35 mm) [3,7], secondary to extra-articular pathologies, which were confirmed by clinical assessment and imaging. This level of reduction was associated with significant functional impairment in daily activities, such as eating, speaking, and maintaining oral hygiene. Cases with intra-articular causes of trismus or incomplete medical records were excluded from the study.

Demographic details, clinical presentation, radiological findings, type of pathology, surgical approach performed, and postoperative outcome in mouth opening (interincisal distance) were available from the records. All patients underwent diagnostic imaging (CT or MRI) and received personalized surgical management depending on their specific diagnosis.

The study complied with the ethical principles of the Declaration of Helsinki (1964) (revised 2024).

Case 1

A 48-year-old male patient was referred to the Department of Oral and Maxillofacial Surgery with a complaint of progressive limitation of mouth opening for the past six months. The main complaint of the patient was recurrent difficulty in eating, which significantly affected his daily life. Past medical history was unremarkable, and the patient had experienced no previous trauma to the oral or maxillofacial region, nor did he report any related symptoms, including pain, swelling, or joint noises. Clinical examination revealed a maximum interincisal mouth opening of 19 mm, which is less than normal. No other abnormalities were found in the intraoral examination with respect to the mucosa (i.e., no lesions or masses) or signs of infection.

**Figure 1:**
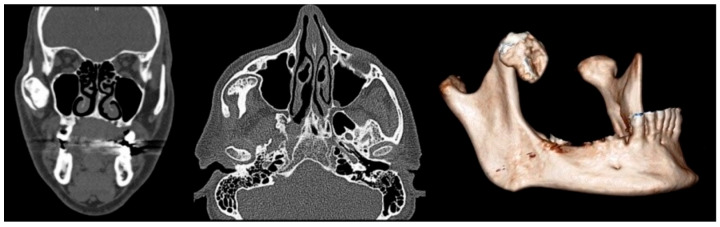
Coronal (**left**), axial (**center**), and 3D reconstructed (**right**) CT images demonstrating an enlarged and elongated right coronoid process.

A panoramic radiograph was performed to investigate the limited mouth opening, which showed a well-defined radiopaque mass at the right coronoid process. A computed tomography (CT) scan was performed to further characterize the lesion and its relationship with surrounding structures. CT images showed an enlarged and elongated right coronoid process with its superior margin extending posteriorly and directly posterior to the zygomatic bone (Figure 1). This abnormal positioning indicated a mechanical obstruction in jaw movement, reducing mouth opening. Based on the imaging findings and the clinical history of slow symptom progression, a provisional diagnosis of a benign osseous neoplasm, likely an osteoma of the right mandibular coronoid process, was made.

Due to a marked restriction of mouth opening, a surgical reconstruction was planned after detailed preoperative planning. The procedure was conducted under general anesthesia with nasotracheal intubation for an unobstructed surgical field. An intraoral approach, combined with an extraoral approach, was deemed appropriate to access the lesion and obtain complete excision of the affected coronoid process.

The intraoral approach began with a similar incision to that performed for the sagittal split osteotomy. This technique offered direct access to the coronoid process. The periosteum and masseter muscle were meticulously elevated on the ascending ramus of the mandible to provide access to the coronoid process. For coronoid process detachment, the temporalis muscle insertion along the anterior border of the ramus was released, and the tendon was dissected and separated from the coronoid process. One channel retractor was introduced into the sigmoid notch to stabilize the field, and a ramus clamp was applied to push and secure the coronoid process. A low coronoidectomy was completed using a reciprocating saw from the sigmoid notch to the anterior oblique ridge. Despite these attempts, the coronoid process could not be resected due to the proximity to the zygomatic arch and lack of intraoral access.

**Figure 2:**
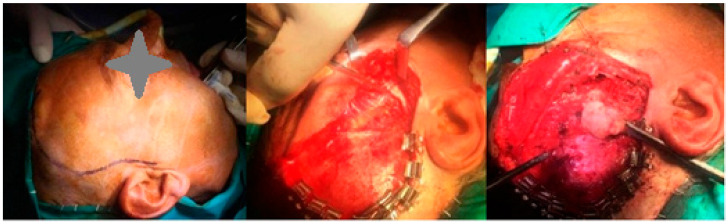
Intraoperative photographs demonstrating the additional extraoral approach. (**Left**) Marking of the hemi-coronal incision with preauricular extension. (**Middle**) Dissection to expose the zygomatic arch. (**Right**) Direct visualization and access to the coronoid process through the extended approach, allowing improved surgical access due to intraoperative limitations encountered during the initial phase.

Given the intraoperative difficulties, an additional extraoral approach was undertaken. Direct access to the zygomatic arch was achieved with a hemi-coronal incision with preauricular extension (Figure 2). Following the elevation of the periosteum, osteotomy of the zygomatic arch was performed at two different points to obtain broad access to the coronoid process. Prior to the osteotomy, a 1.5 mm titanium plate was placed to stabilize the anatomical positioning of the arch during the postoperative period. Once the coronoid process was fully exposed, it was successfully removed. The zygomatic arch was then repositioned anatomically and stabilized using the preplaced titanium plate.

**Figure 3:**
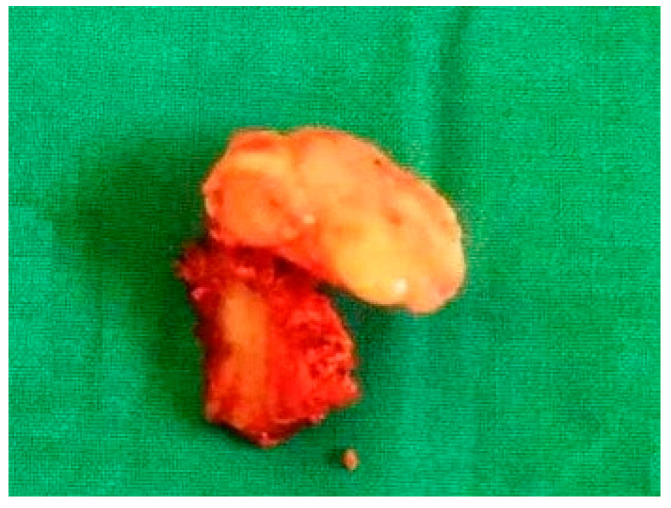
The resected specimen shows a bony, mushroom-shaped mass.

The resected specimen was a bony mushroom-shaped mass (approximately 2.5 cm × 3 cm × 3 cm) (Figure 3). Histopathological examination of the lesion showed a highly cellular collagenized stroma in continuity with interspersed bony trabeculae. At higher magnification, cancellous lamellated bone was evident, with lacunae containing osteocytes and osteoblastic rimming. These findings were consistent with the diagnosis of an osteoma, a benign bone-forming tumor.

**Figure 4:**
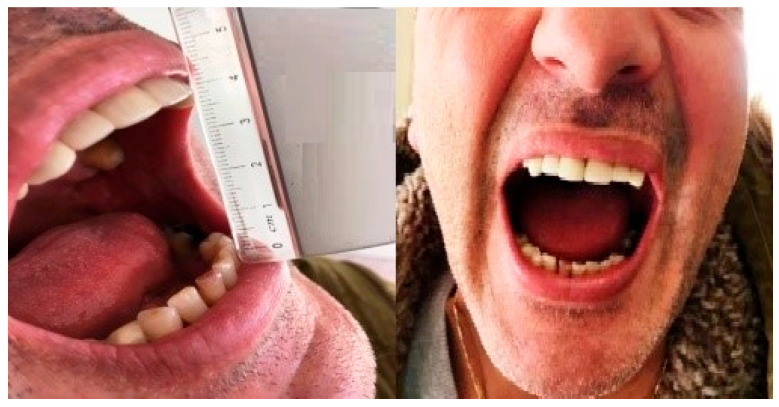
Postoperative clinical photographs showing improved mouth opening. (**Left**) Measurement of interincisal distance at three-month follow-up demonstrates an opening of 39 mm. (**Right**) One-year follow-up confirms stability of surgical results with maintained mouth opening and no signs of recurrence or functional limitation.

The postoperative recovery was uneventful, with no immediate complications like infection, nerve injury, or malocclusion. The patient showed a marked improvement in mouth opening, which increased to 36 mm. He was also counseled to initiate physiotherapy and mouth-opening exercises to improve the function of the mandible and to prevent fibrosis postoperatively. At the three-month follow-up, the patient’s mouth opening improved to 39 mm (Figure 4). A 1-year follow-up examination revealed stability of surgical results without signs of recurrence or functional impairment.

Case 2

A 10-year-old female patient was referred to the Department of Oral and Maxillofacial Surgery by her pediatrician due to a progressive limitation in mouth opening. The patient had difficulty with activities like eating and speaking without any associated pain. Intraoral examination showed a maximum interincisal opening (MIO) of 17 mm, which was remarkably limited for her age. Her medical history was unremarkable, and no injuries, infections, or systemic illnesses were documented. There was no family history of similar symptoms, and an examination of the temporomandibular joints showed no sounds, pain, or signs of dysfunction.

**Figure 5:**
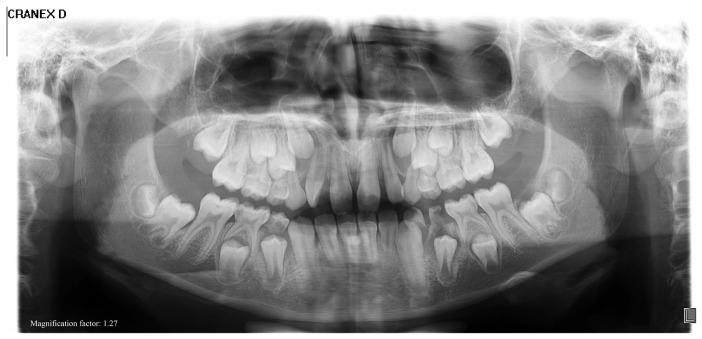
Initial panoramic radiograph showing bilateral elongation of the coronoid processes. The structural abnormality is evident on both sides and correlates clinically with the patient’s restricted mouth opening.

Initial panoramic radiography showed bilateral elongation of the coronoid processes (Figure 5), indicating structural abnormalities as the cause of her restricted mouth opening.

**Figure 6:**
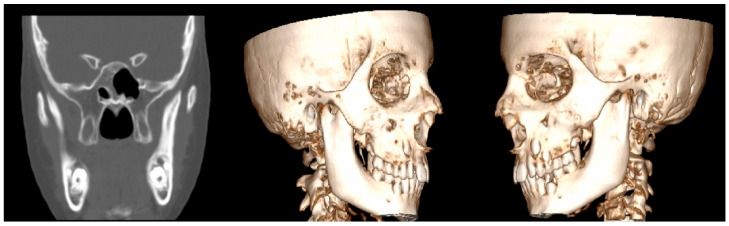
Coronal CT slice (**left**) and 3D reconstructed views (**center** and **right**) demonstrating bilateral elongation of the coronoid processes. Additionally, heterotopic bone formation is visible on the medial and inferior surfaces of the bilateral zygomatic arches, contributing to the patient’s limited mouth opening.

Computed tomography (CT) imaging offered enhanced visualization, confirming the elongation of the bilateral coronoid processes and revealing the presence of heterotopic bone on the medial and inferior surfaces of the bilateral zygomatic arches (Figure 6). These findings indicated mechanical interference between the coronoid processes and the zygomatic arches during mandibular movement. Based on the clinical and imaging findings, as well as the history of slow symptom progression, a diagnosis of coronoid impingement syndrome (CIS) was established.

**Figure 7:**
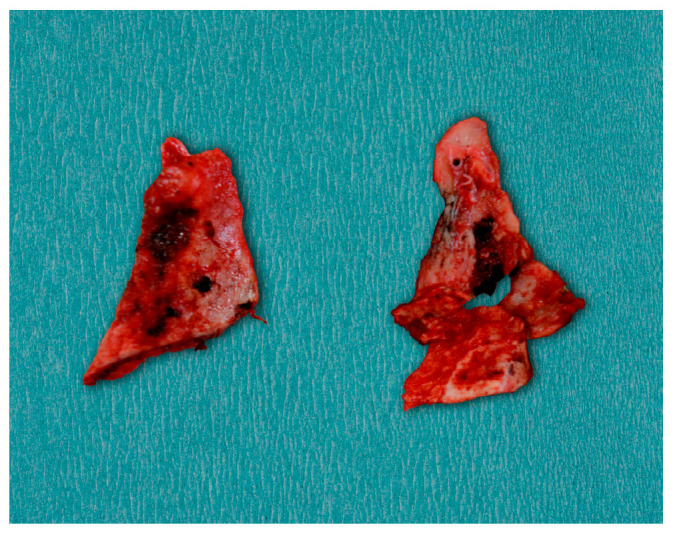
Resected bilateral coronoid processes.

Surgical treatment was planned in order to restore functional mouth opening. Nasotracheal intubation was expected to be challenging because of the significant restriction of the mouth opening. General anesthesia was achieved, and nasotracheal intubation was conducted with a fiberoptic scope because the head-scope-guided approach was impossible. An intraoral approach was used for the bilateral coronoidectomies to reduce external scarring and postoperative morbidity. Bilateral buccal mucosa incisions at the level of the ascending ramus of the mandible were made to expose the coronoid processes. The periosteum and surrounding soft tissues were elevated, exposing the elongated coronoid processes. Performing the procedure with a reciprocating saw, the coronoid processes were resected (Figure 7) entirely on both sides to remove mechanical interference with the zygomatic arches. Adequate hemostasis was obtained during the procedure.

Immediately following surgery, the operating room’s maximal interincisal opening increased to 37 mm. This immediate improvement confirmed the restriction’s mechanical nature and the intervention’s success. Postoperative recovery was uneventful, without any complications. The patient was discharged with instructions to begin a structured physiotherapy program, including mouth-opening exercises, starting four weeks postoperatively to prevent fibrosis and maintain functional improvement.

At the ten-month follow-up, the maximal interincisal opening had improved to 45 mm, reflecting sustained functional gains. At the one-year follow-up, the patient exhibited stable and satisfactory mouth opening with no signs of recurrence or postoperative complications. The surgical outcome was highly successful, and the patient had fully recovered.

Case 3

**Figure 8:**
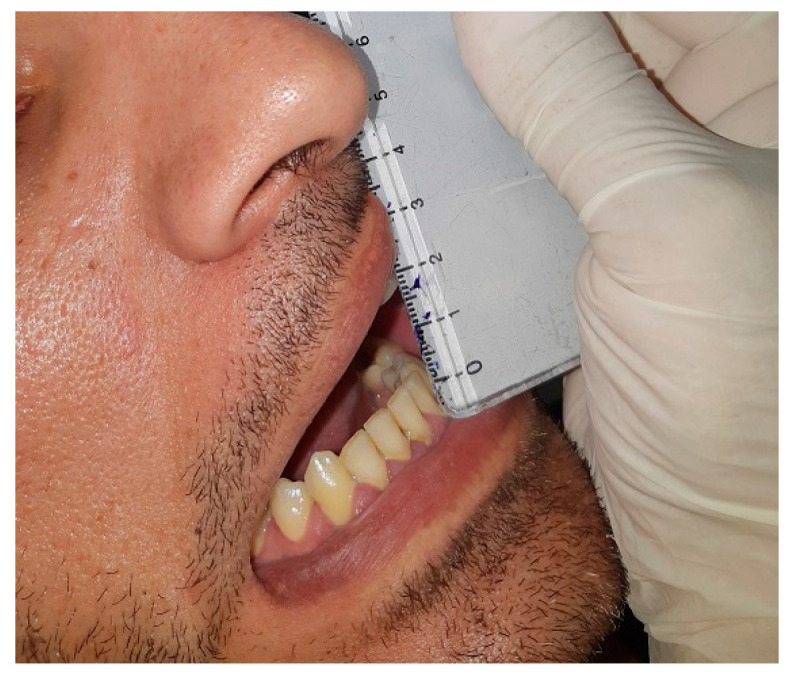
Preoperative clinical photograph showing a maximal incisal opening (MIO) of 15 mm, indicating a significant limitation in mouth opening.

A 34-year-old male patient was referred by his dentist to the Department of Oral and Maxillofacial Surgery, complaining of reduced mouth opening that had started about a month earlier. The clinical examination showed a maximal incisal opening (MIO) of 15 mm (Figure 8). The patient’s medical history was notable for a facial injury on the right side sustained three months prior. There was no past medical history of systemic disease or other pertinent conditions.

**Figure 9:**
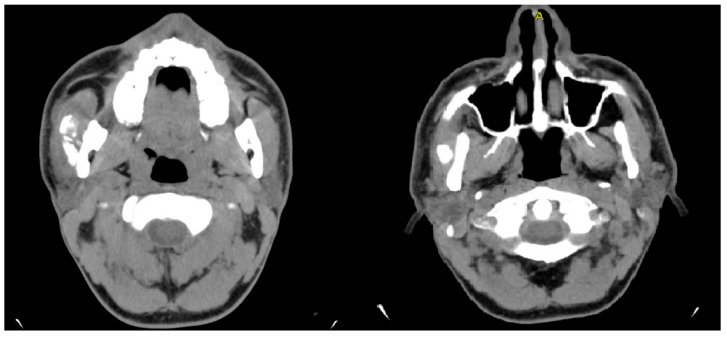
Axial CT images revealing a well-defined calcified mass within the right masseter muscle.

Imaging studies were performed to assess the restricted mouth opening. Orthopantomography and computed tomography (CT) showed a well-defined calcified mass in the right masseter muscle (Figure 9). There was no evidence of fractures or abnormality of the cranial visceral bones. Laboratory tests showed normal serum calcium, phosphorus, and parathyroid hormone levels, excluding systemic metabolic causes of calcification. Given the clinical history and imaging findings, along with the background of previous trauma with gradual symptom development, a provisional diagnosis of myositis ossificans traumatica (MOT) was made.

Surgical intervention was indicated, and the patient was transferred to the operating room. Under general anesthesia, fiberoptic-assisted nasotracheal intubation was performed owing to the limited opening of the mouth. The calcified mass in the right masseter muscle was excised through the intraoral approach to avoid excessive visible scarring. The lesion was circumscribed and measured 2.6 cm at its largest diameter, allowing for excision. Histopathological examination showed alternating lamellar bone with fat cells, fibrous tissue, and thin-walled vascular spaces, consistent with a diagnosis of MOT.

The patient had an uneventful immediate postoperative recovery without any complications. Upon discharge, the MIO had improved to approximately 34 mm. A regimen of forced physical therapy and mouth-opening exercises was recommended to prevent fibrosis and maintain an improved range of motion.

**Figure 10:**
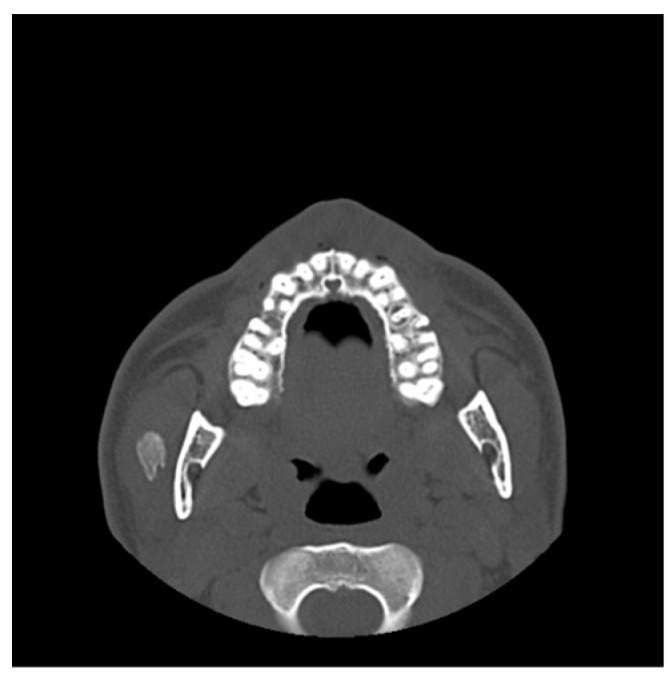
An axial CT scan showed a well-defined calcified lesion within the left masseter muscle, anatomically distinct from the previous surgical site. The radiologic appearance is consistent with recurrent or new-onset myositis ossificans.

Two months after surgery, the patient returned to the department complaining of a new reduction in mouth opening. Clinical examination revealed an MIO of 20 mm. Imaging studies, including CT scans, identified a well-defined calcified lesion within the left masseter muscle, distinct from the previous surgical site (Figure 10). Given the recurrence of symptoms, a second surgical intervention was planned. Surgery was performed under general anesthesia with fiberoptic-assisted nasotracheal intubation, and the calcified mass in the left masseter muscle was excised by the intraoral approach.

Histological analysis of the excised lesion described a central zone of bone tissue with abundant osteoblasts surrounded by mature bone, findings consistent with myositis ossificans. The edges of the specimen were free of tumor or other pathological findings, ensuring complete excision.

**Figure 11:**
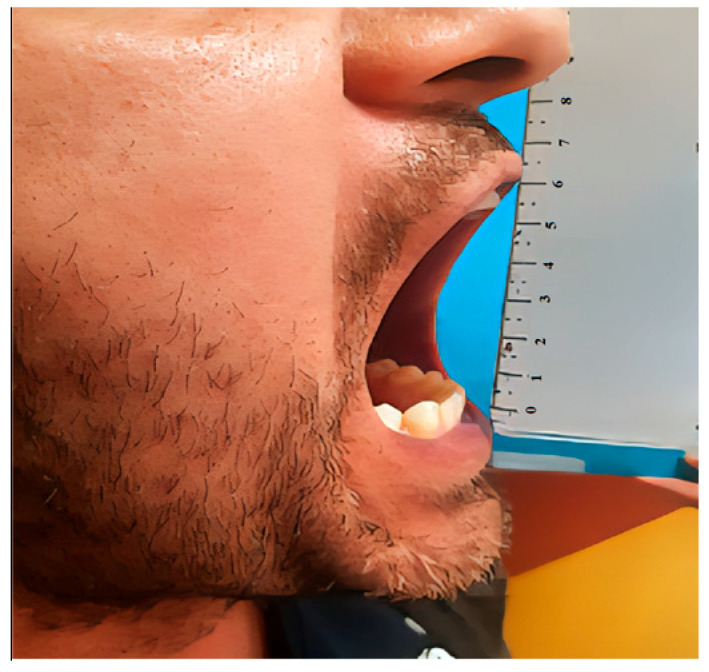
Clinical photograph at six-month follow-up showing a maximal incisal opening (MIO) of 52 mm, demonstrating excellent functional recovery and restoration of normal mandibular mobility.

The postoperative course was again uneventful, and the patient demonstrated a significant improvement in mouth opening, with an MIO of 36 mm achieved soon after surgery. He was once more advised to perform mouth-opening exercises and continue physiotherapy. At the six-month follow-up, the patient’s MIO had increased to 52 mm (Figure 11), reflecting excellent functional recovery. At the one-year follow-up, the patient remained asymptomatic, with no signs of recurrence and stable mouth-opening capacity, indicating a successful surgical outcome and long-term resolution of the condition.

Case 4

A 44-year-old healthy male patient was referred to the Craniomaxillofacial Surgery Department by his physiotherapist due to severe restriction of mouth opening. The patient reported a history of extraction of an impacted mandibular third molar (#38) three years prior. According to the patient, the extraction was straightforward and without immediate complications. As per his medical history, the onset of reduced mouth opening occurred suddenly and without apparent cause. Initial clinical and imaging evaluations by the patient’s dentist prompted a referral to a specialized physiotherapy center within our hospital.

Despite undergoing nine physiotherapy sessions, there was no improvement in the patient’s condition. During this period, the patient developed episodic intermittent hypoesthesia of the left inferior alveolar nerve. Given the inadequate response following therapy, he was referred to our clinic for further evaluation and management. On presentation, the patient exhibited no abnormal swelling, facial asymmetry, or hypertrophy of the masticatory muscles. The temporomandibular joint (TMJ) showed no frictional sounds, and no compression pain was elicited upon palpation of the joint areas bilaterally. The maximal incisal opening (MIO) was limited to 13 mm, and the posterior dental contacts were symmetrical.

**Figure 12:**
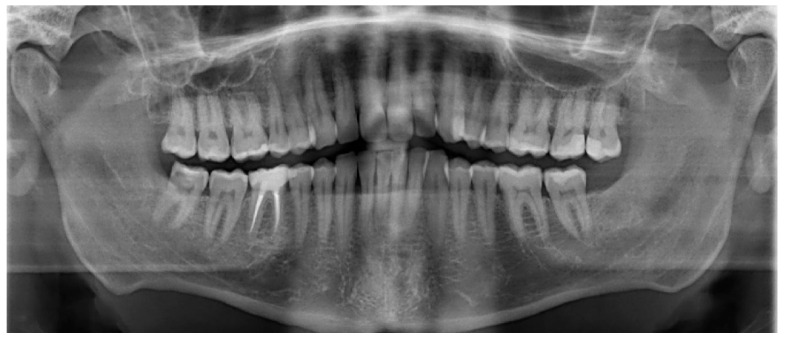
Postoperative panoramic radiograph showing normal bilateral temporomandibular joint (TMJ) articulation. No signs of odontogenic infection, recurrence, or structural abnormalities are present.

A panoramic radiograph demonstrated normal TMJ articulation bilaterally, with no evidence of odontogenic infection or structural abnormalities (Figure 12). Magnetic resonance imaging (MRI) revealed mild bilateral anterior disc dislocation with arthropathy, more pronounced on the left side, and incomplete translation of the disc on mouth opening. No intracranial pathology or secondary cause of head and facial pain was identified. A computed tomography (CT) scan ruled out any pathological findings in the mandible.

**Figure 13:**
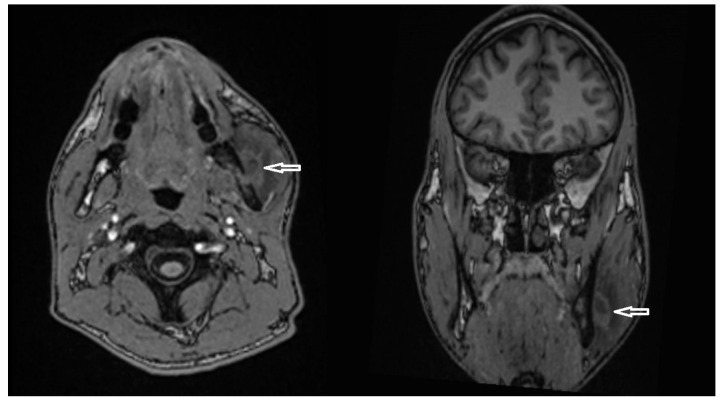
Axial (**left**) and coronal (**right**) T1-weighted MRI images showing a hyperintense lesion adjacent to the lateral aspect of the distal mandibular ramus (white arrows), suggestive of a developing abscess.

Based on the findings, a provisional diagnosis of tendomyopathy of the masseter muscles was established. The patient was started on tizanidine (4 mg) and re-referred for ongoing physiotherapy. After 1 week, the patient stopped taking the medication because of dizziness and muscle limpness. He also did not want to continue with physiotherapy. Tolperisone (150 mg) replaced tizanidine, which slightly improved trismus. Three months later, the patient presented with worsening symptoms. Mouth opening had further reduced to 2 mm, and he presented with swelling in the region of the left mandibular angle. The hypoesthesia of the inferior alveolar nerve, which had been intermittent, was now persistent. The patient was afebrile and reported no difficulty swallowing. An MRI revealed findings suggesting a developing abscess adjacent to focal cortical defects on the lateral side of the distal mandibular ramus, with possible incipient abscess formation medially (Figure 13). The masticatory musculature was associated with diffuse edematous or phlegmonous swelling, particularly the masseter and medial pterygoid muscles on the left side. The distal mandibular ramus also showed an increased bone marrow signal, corresponding to acute osteomyelitis.

Surgical management by incision and drainage of the abscess and surgical curettage was performed under local anesthesia. Gram stain, culture, and sensitivity testing of swabs taken during the procedure showed a polymicrobial anaerobic population primarily composed of the *Streptococcus milleri* group. The histopathological study showed foci of chronic inflammatory infiltration and medullary fibrosis, leading to the diagnosis of late-diagnosed chronic osteomyelitis of the mandible. Pharmaceutical management consisted of a combination of amoxicillin (875 mg)/clavulanic acid (125 mg) and ibuprofen (600 mg) for anti-inflammatory. Fifteen days postoperatively, during a follow-up examination, the patient presented with a marked mouth opening measuring 39 mm, lower swelling in the mandibular angle region, and significant pain relief.

**Figure 14:**
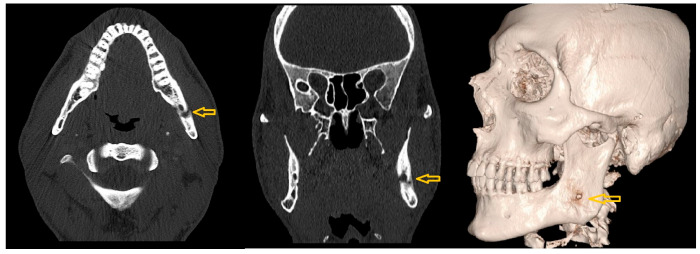
Axial (**left**), coronal (**middle**), and 3D reconstructed (**right**) CT images showing a localized area of osteolysis in the left mandibular body at the level of the mandibular canal (yellow arrows).

Further CT imaging disclosed a localized osteolysis area in the left mandibular corpus at the height of the mandibular canal (Figure 14), along with slight bony dehiscence of the medial and lateral compacta. No residual or adjacent soft tissue swelling was evident. The antibiotic was continued for another 15 days, and the patient showed marked improvement. At the end of treatment, the patient showed a mouth opening of 48 mm and remained pain-free.

Case 5

A 45-year-old female patient was referred to the Craniomaxillofacial Surgery Department by the otorhinolaryngology (ENT) team with severe restriction of mouth opening and temporomandibular joint (TMJ) dysfunction. The patient presented with left otalgia and reduced mouth opening for three days and was initially diagnosed with otitis externa. Her symptoms persisted despite being treated with local antibiotic drops, leading to further evaluation. On examination, there was no evidence of swelling, erythema, or fluctuation near the TMJ. Severe tenderness was noted over the left TMJ. There was the restriction of maximal incisal opening (MIO) to 10 mm with right-sided mandibular deviation on occlusion. There were no visible mucosal abnormalities or evidence of odontogenic infection.

**Figure 15:**
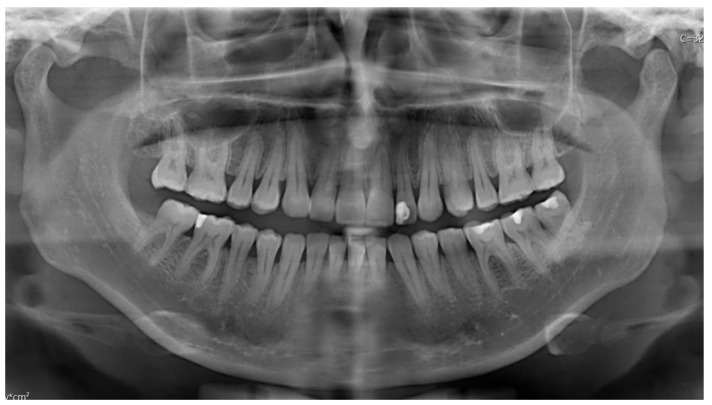
Panoramic radiograph demonstrating normal bilateral temporomandibular joint (TMJ) articulation. No evidence of odontogenic infection, pathological lesions, or structural abnormalities is observed.

Panoramic radiographs indicated bilateral normal TMJ articulation without signs of odontogenic infection or structural changes (Figure 15).

**Figure 16:**
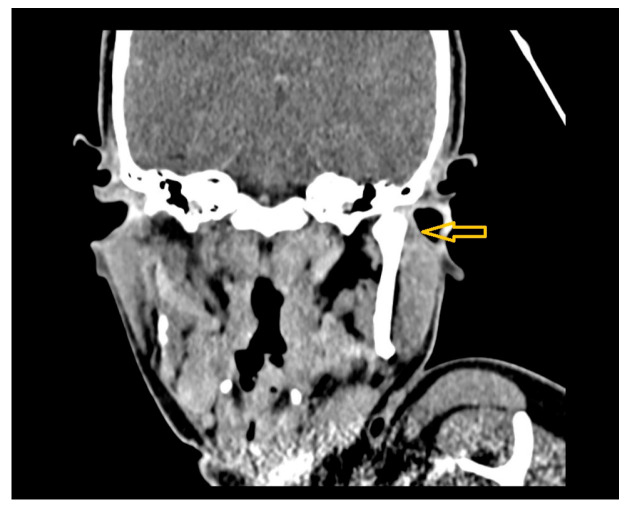
Coronal CT image showing subluxation of the left temporomandibular joint (TMJ), accompanied by surrounding soft tissue swelling extending toward the external auditory canal (yellow arrow).

A CT scan shows subluxation of the left TMJ (Figure 16), with soft tissue swelling up to the external auditory canal, but no evidence of abscess or joint arthrosis was found.

**Figure 17:**
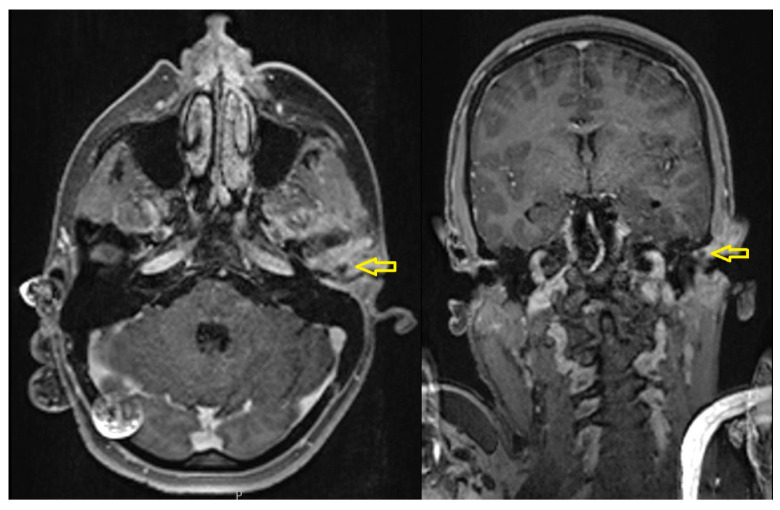
Axial (**left**) and coronal (**right**) contrast-enhanced MRI images showing effusion in the left temporomandibular joint (TMJ) and surrounding phlegmonous inflammatory changes (yellow arrows).

Magnetic resonance imaging (MRI) revealed TMJ effusion with phlegmonous changes posterior to the mandibular condyle and a small suspected abscess measuring 4 × 5 mm (Figure 17).

**Figure 18:**
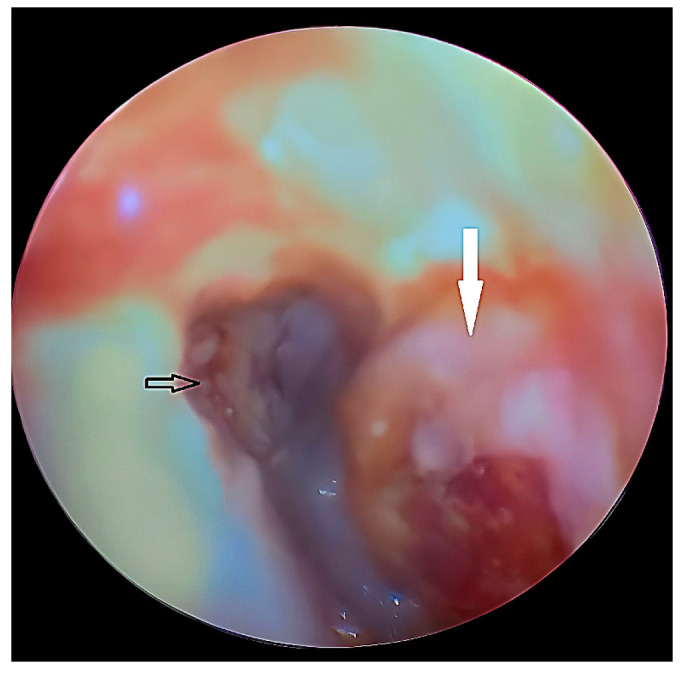
Otoscopic view showing the tympanic membrane (black arrow) and a bulging abscess (white arrow) in the anterior wall of the external auditory canal.

Surgical intervention was performed under local anesthesia, including incision and drainage of an abscess in the anterior external ear canal wall (Figure 18), followed by placement of a medicated Meche with gentamicin cream. Systemic antibiotics were initiated with Co-Amoxicillin. Swabs collected during the procedure revealed methicillin-resistant *Staphylococcus aureus* (MRSA), prompting a switch to Clindamycin (600 mg) thrice daily.

Gradually, over the subsequent weeks, the patient’s pain intensity decreased, and mouth opening improved to 25 mm. At 15 days postoperatively, the patient had marked improvement in pain, and mouth opening had improved to 42 mm. She was advised to perform mouth-opening exercises and continue physiotherapy. Mouth opening was further improved at the end of treatment to 51 mm while the patient remained pain-free with no evidence of residual infection or complications.

## 3. Results

Five rare causes of extra-articular trismus were included. The pathologies were coronoid process hyperplasia and osteoma, myositis ossificans traumatica, chronic suppurative osteomyelitis of the ramus mandible, and deep-space abscess originating from the external auditory canal. Detailed diagnosis was made through various high-resolution imaging modalities, including computed tomography (CT) and magnetic resonance imaging (MRI), to localize and characterize the lesions appropriately. Surgical management was guided by each case’s etiology and anatomical complexity and included coronoidectomy, local excision of ossified or infected tissue, and drainage of deep-seated abscesses. All five patients in this study showed significant improvement in mouth opening following surgery, with the interincisal distances increasing from less than 20 mm before the operation to more than 35 mm after it. Structured physiotherapy and regular follow-up further strengthened this functional recovery to achieve gradual and sustained recovery (Table 1).

## 4. Discussion

Trismus, known as “locked jaw,” is frequently encountered in oral and maxillofacial surgery and can have a devastating impact on the quality of life of a patient [9,11]. Although the normal range of interincisal distance varies among individuals and has been found to be between 40 and 60 mm [9,11], any significant decrease in this should be considered as a possible underlying disease and should be treated accordingly [12].

Trismus has a broad etiologic spectrum, which can be classified as intra-articular and extra-articular [1,9,10]. Intra-articular causes include pathologies of the temporomandibular joint (TMJ) itself, such as ankylosis, arthritis, synovitis, and meniscal derangements. These include several structural abnormalities or inflammatory processes directly affecting the joint [13]. The extra-articular causes, however, are numerous and may affect the adjacent structures, such as the masticatory muscles, coronoid processes, and soft tissues of the oral cavity [14,15]. These can be categorized further into congenital [16] and acquired conditions, with the acquired causes typically being trauma [17,18], infection [19,20], tumors [21,22], and fibrotic changes [23,24]. The table below (Table 2) summarizes the most common causes for trismus based on accepted classifications.

Less commonly, trismus could be caused by tumors that involve the coronoid process of the mandible [25]. Notably, osteomas, benign bone tumors, can develop in this area and lead to restricted mouth opening by physically preventing mandibular movement against the zygomatic arch [26]. To date, only a few cases of coronoid osteoma have been reported in the literature since Lewars’ first report in 1959 [27]. Petronis et al. (2024) recently addressed the difficulty in diagnosing coronoid osteomas because they are often asymptomatic in the early stages [28]. These tumors are generally diagnosed by clinical examination and imaging studies, including computed tomography (CT) and magnetic resonance imaging (MRI), supported by histopathological evaluation [29,30,31]. In our patient, the tumor was classified as a peripheral and cancellous variant of osteoma, originating from the periosteum of the coronoid process. Notably, no association with Gardner’s syndrome [32]—identified by colorectal polyposis, skeletal abnormalities, and supernumerary teeth—was recognized in this patient.

Osteomas usually appear in adults older than 40 years and show a male predominance (a male-to-female ratio of approximately 2:1) [33]. The pathogenesis of etiology is still a matter of debate. Some investigators regard οsteomas to be true neoplasms, while others believe they are developmental anomalies or reactive lesions in response to trauma or infection [31]. However, in the absence of trauma or infection, as in our case, the etiology is unknown. Differential diagnoses for osteomas are osteochondroma, fibrous dysplasia, Paget’s disease, and ossifying fibroma [34,35]. They are generally benign and asymptomatic, but the surgery is often indicated if they cause functional disturbance, as in our patient who underwent intraoral coronoidectomy. This approach minimizes scarring and postoperative morbidity [35]. At the one-year follow-up, the patient had fully recovered without any recurrence.

Another rare etiology of trismus is bilateral coronoid process hyperplasia [36]. This condition, which is the exception, is seen in approximately 5% of mandibular hypomobility cases and is described by the abnormal elongation of the coronoid processes [37,38]. This elongation protrudes against the temporal surface of the zygomatic bone or the medial surface of the zygomatic arch while moving the mandible [39,40]. According to the study by Goh et al. (2020), most cases occur in men, and the average age of onset is 23 years [39]. However, it is rare in children under the age of 10 [40]. Our case of a 10-year-old female patient with bilateral coronoid process hyperplasia underscores the progressive and painless nature associated with the condition. Reduced mouth opening can be insidious, often undetected, until the function is seriously impaired and identified, in this case, during a routine pediatric check-up.

Coronoid hyperplasia has an obscure etiology. Possible explanations include genetics, trauma, overactivity of the temporalis muscle, and hormone ratios [41,42]. However, none of these factors were found in our patient. The most common treatment is to surgically remove the coronoid processes by performing a coronoidectomy through intraoral or extraoral approaches [43]. Intraoral coronoidectomy is often preferred due to its lower risk of complications, like facial nerve injury and external scarring [43]. These findings are consistent with those reported by Nogueira et al. (2015) in their retrospective analysis of similar cases [44]. The authors emphasized the importance of early intervention and physiotherapy. Postoperative physiotherapy is important to maintain an adequate range of motion and prevent recurrence [45,46]. In our case, the patient also reported considerable improvement and absence of recurrence at the one-year follow-up. 

Myositis ossificans traumatica (MOT) is another rare etiology of trismus. MOT is described as the ectopic ossification of skeletal muscle and usually occurs after trauma [47]. Although it most often presents in the quadriceps or brachialis muscles, the masseter muscle in the head and neck region is not an unprecedented involvement [48]. Similar cases have been reported in the literature [49,50,51,52] discussing the diagnostic and therapeutic dilemmas of MOT. Trauma is a commonly accepted primary precipitating factor, although the exact pathogenesis of MOT is unclear. Proposed mechanisms include migrating osteoprogenitor cells to soft tissues and detaining periosteal fragments or differentiated cells exposed to bone morphogenic proteins [53]. Histopathological assessment of MOT lesions commonly indicates a three-zone arrangement, encompassing a peripheral layer of mature lamellar bone, an intermediate zone of immature osteoid and cartilage, and a central zone of granulation tissue and muscle necrosis [48].

In our case, a patient presented with trismus following trauma to the right side of the face. Imaging studies showed a calcified mass in the masseter muscle, and MOT was confirmed histopathologically. Subsequently, the lesion was surgically excised, and intensive physiotherapy was pursued. However, the patient experienced recurrence two months later, necessitating a second surgical intervention. This fact underscores the difficulty in managing MOT, including the best time for surgical intervention and how to maximize compliance with postoperative physiotherapy to prevent recurrence. Jović et al. (2021) also reported a high recurrence rate in cases of incomplete excision or insufficient rehabilitation [50]. Other treatments for MOT, including non-steroidal anti-inflammatory drugs, bisphosphonates, and low-dose radiation therapy, have also been investigated, but the evidence of their effectiveness is inconsistent [47,48,49,50,51,52,53,54].

Chronic osteomyelitis of the mandible is a challenging and often underdiagnosed condition that can significantly impact a patient’s quality of life, particularly when diagnosed late [55]. Its insidious onset and nonspecific symptoms (pain, swelling, and restricted mouth opening) make an early diagnosis and treatment difficult [56]. In our case, a patient was presented initially with unexplained trismus and intermittent hypoesthesia of the inferior alveolar nerve, which are both red flags for more profound pathological processes. Over time, the disease progressed to include swelling, abscess formation, and cortical bone defects on imaging modalities. In the current case, magnetic resonance imaging (MRI) was instrumental in demonstrating inflammatory changes in the masticatory muscles and changes in the mandibular bone marrow signal that suggest acute-on-chronic osteomyelitis [57]. These results, together with findings from CT imaging, provide further evidence that comprehensive imaging plays an important role in the diagnosis and management of patients with mandibular osteomyelitis. The delay in diagnosis that is common in such cases illustrates the need for osteomyelitis to be part of the differential diagnosis in people who present with progressive trismus, once more common causes have been excluded [56].

Management of chronic osteomyelitis requires a multimodal approach. Surgery seeks to contain the infection and eliminate sources of persistent inflammation [58]. In this case, surgical management encompasses the exact methods for draining abscesses and curettage of the involved area, all of which match standard practices, such as sequestrectomy, saucerization, and decortication. Resection and subsequent reconstruction may be indicated in refractory cases [59]. The microbiological analysis in our case identified *Streptococcus milleri* as the predominant organism. This abscess-forming pathogen belongs to the Streptococcus anginosus group and is characterized by its tendency for deep infections, such as osteomyelitis [60]. Identification of *S. milleri* guided antibiotic therapy, which was critical in the eventual resolution of infection.

This case illustrates the importance of a high index of suspicion for chronic osteomyelitis in patients presenting with progressive trismus and nonspecific symptoms. Trismus is a clinical entity that needs a comprehensive diagnostic workup involving clinical assessment and advanced imaging. Panoramic radiography, CT, and MRI are important for detecting the underlying cause and for treatment planning. Physiotherapy following surgery is vital for the return of mandibular function and prevention of recurrence. Imaging studies, like open-mouth panoramic radiography, should be repeated periodically to follow recovery with time [61,62].

External auditory canal abscess or inflammation can be a rare but clinically significant cause of temporomandibular joint (TMJ) dysfunction and trismus [63]. Both structures are susceptible to secondary involvement due to their close anatomical relationship, with the external auditory canal neighboring the mandibular condyle. Infection in the external auditory canal can directly extend to the TMJ or compress adjacent tissues as a result of abscess expansion, resulting in pain, decreased movement, and dysfunction of the jaw [64]. Such cases often require advanced imaging to assess the extent of the pathology and ascertain the diagnosis to allow for appropriate management [64,65].

In the presented case, microbiological analysis revealed methicillin-resistant *Staphylococcus aureus* (MRSA) as the causative pathogen. MRSA’s virulent behavior, resistance mechanisms, and association with healthcare settings are well described. The ability to create biofilms and infiltrate host tissues makes treatment challenging, often necessitating a dual method of surgical drainage and specific antibiotic therapy [66]. In our case, MRSA identification after the first 48 h of the Co-Amoxicillin led to changing the antibiotic to Clindamycin; this choice is a frequent option for its excellent effectiveness against MRSA and good penetration in bone and soft tissues. Therefore, this specific antimicrobial approach, together with immediate surgical treatment, is necessary to control the infections of the TMJ and adjacent parts [67].

Imaging was crucial to exposing the extent of the disease. Computed tomography (CT) demonstrated subluxation of the TMJ with surrounding soft tissue swelling. At the same time, magnetic resonance imaging (MRI) showed joint effusion, phlegmonous change, and a small abscess adjacent to the anterior wall of the external auditory canal, with no abnormality noted on panoramic radiographs. This emphasizes the role of CT and MRI in head and neck infections with complex inflammatory anatomical microenvironments [65].

Management consisted of incision and drainage of the abscess, followed by medicated dressing and systemic antibiotics. That approach effectively reduced infection and restored function. The resolution of the symptoms and the improvement in maximal incisal opening to 51 mm at the end of the treatment emphasize the effectiveness of early aggressive intervention.

Rehabilitation of trismus is the major phase after its treatment, and it requires a multidisciplinary approach to manage and achieve optimal recovery along with comprehensive prevention of future recurrences. These multidisciplinary teams usually consist of oral and maxillofacial surgeons, physiotherapists, and speech therapists, all of whom are integral in managing the functional and structural consequences of trismus [68].

The mainstay of rehabilitation is physiotherapy, which consists of active and passive stretching exercises to enhance the range of mandibular movement progressively [69]. These exercises are normally augmented through the use of dynamic devices, similar to continuous passive motion machines, and specialized implements, together with bite blocks, jaw stretchers, or mouth screws [70]. Individualized splints are specialized aids that help apply uniform lengthening to the masticatory muscles and prevent re-apposition. Regular physiotherapy and following a prescribed routine are essential for having a better recovery, and relapse can be prevented as it tends to fix the recovery in the jaw. For certain conditions, such as hyperplasia of the coronoid process or myositis ossificans, early initiation of physiotherapy—often immediately after surgical fixation—has been shown to improve outcomes. Early mobilization preserves the functional mobility of the temporomandibular joint and surrounding structures and prevents postoperative fibrosis or scarring, limiting recovery [68,70].

Speech therapy may then also be initiated, especially when trismus impairs swallowing, speech articulation, or oral functions. A holistic approach that covers functional and mechanical aspects while rehabbing a patient from trismus is vital to ensure the patient regains function and quality of life [71].

## 5. Conclusions

In conclusion, trismus may result from various aetiologies, including benign osteomas, coronoid process hyperplasia, myositis ossificans, and chronic osteomyelitis. The cases presented in this study emphasize the clinical complexity associated with such rare extra-articular causes and highlight the importance of early diagnosis, multidisciplinary evaluation, and customized surgical planning. Early intervention and structured postoperative care are essential in achieving functional outcomes. Although the results are derived from selected clinical scenarios, these findings report pivotal management areas and advocate the requirement for continued research to improve diagnostic care, develop surgical interventions, and standardize rehabilitation protocols to improve patient management.

## Figures and Tables

**Table 1:** Patients with extra-articular causes of trismus.

	Sex	Age	Etiology	Imaging	Preoperative Interincisal Distance	Operative Intervention	Postperative Interincisal Distance
1	Male	48	Osteoma of the coronoid process	OPG CT	19 mm	Coronoidectomy	39 mm
2	Female	10	Bilateral hyperplasia of the coronoid processes	OPG CT	17 mm	Bilateral coronoidectomy	45 mm
3	Male	34	Myositis ossificans traumatica	CT	15 mm	Excision of calcified mass	52 mm
4	Male	44	Chronic osteomyelitis of the mandible	OPG CT/MRI	13 mm	Incision and drainage of the abscess and surgical curettage	48 mm
5	Female	45	External auditory canal abscess	OPG CT/MRI	10 mm	Incision and drainage of the abscess	51 mm

**Table 2:** Patients with extra-articular causes of trismus.

Category	Etiology	Examples
Intra-articular	Structural abnormalities or inflammation	TMJ ankylosis, arthritis, synovitis, Discus pathology
Extra-articular	Neoplastic	Osteoma, sarcoma, lymphoma
	Traumatic	Myositis ossificans, mandibular fractures
	Infectious	Peritonsillar abscess, mandibular osteomyelitis
	Fibrotic	Submucous fibrosis, radiation-induced fibrosis
	Neurological	Tetanus, dystonia
	Iatrogenic	Postoperative complications, TMJ surgeries

## Data Availability

The data presented in this study are available on request from the corresponding author.

## Abbreviations

The following abbreviations are used in this manuscript:

CT	Computed tomography
MRI	Magnetic Resonance Imaging
OPG	Orthopantomogramm
TMJ	Temporomandibular joint
MIO	Maximum interincisal opening
CIS	Coronoid impingement syndrome
MOT	Myositis ossificans traumatica
MRSA	Methicillin-resistant *Staphylococcus aureus*
SAG	*Streptococcus anginosus* group
ENT	Otorhinolaryngology (ear, nose, throat)
